# Correlation of Bond Strength and Dentinal Tubule Penetration Evaluation of Four Different Endodontic Sealers: AH Plus, MTA Fillapex, Endoseal MTA, and Endoseal TCS (Maruchi): An In Vitro Study

**DOI:** 10.1155/ijod/9972346

**Published:** 2025-12-17

**Authors:** Arezoo Mirzaei Sadeghloo, Seyedali Seyedmajidi, Akam Saeidi, Elham Mahmoudi

**Affiliations:** ^1^ Student Research Committee, Babol University of Medical Sciences, Babol, Mazandaran, Iran, mubabol.ac.ir; ^2^ Dental Materials Research Center, Health Research Institute, Babol University of Medical Sciences, Babol, Mazandaran, Iran, mubabol.ac.ir; ^3^ Oral Health Research Center, Health Research Institute, Babol University of Medical Sciences, Babol, Mazandaran, Iran, mubabol.ac.ir

**Keywords:** dental bonding, root canal filling materials, root canal sealants

## Abstract

**Background:**

The sealer’s penetration into dentinal tubules creates a physical barrier that entombs any remaining bacteria and increases the surface contact with the root canal walls. This study aimed to evaluate the dentinal tubule penetration and push‐out bond strength of four sealers and to assess the correlation between penetration depth and bond strength.

**Methods:**

In this in vitro study, 40 human single‐rooted anterior teeth were divided into four groups based on the sealer type and obturated using the single cone technique. Three slices of 1 ± 0.1 mm thickness were then prepared from each tooth at three regions—coronal, middle, and apical thirds—perpendicular to the longitudinal axis of the root. The depth and percentage of sealer penetration into the dentinal tubules were measured using confocal laser scanning microscopy (CLSM) on the prepared slices. To assess the bond strength of the sealers to dentin walls, a push‐out bond strength test was performed using a universal testing machine. Data were analyzed using SPSS software through ANOVA, Tukey post hoc and Pearson’s correlation tests. A *p*‐value of <0.05 was considered statistically significant.

**Results:**

Statistically significant differences were observed in the extent and percentage of penetration, and the push‐out bond strength of the studied sealers (*p*  < 0.001 for all). Additionally, a significant correlation was found between bond strength and penetration percentage for AH Plus (*p* = 0.046 and *r* = 0.366), between bond strength and both penetration extent (*p* = 0.013 and *r* = 0.448) and penetration percentage (*p* = 0.001 and *r* = 0.572) for Endoseal MTA, and between bond strength and penetration extent for Endoseal TCS (*p* = 0.001 and *r* = 0.57).

**Conclusion:**

Calcium silicate‐based sealers (Endoseal MTA and Endoseal TCS) achieved the greatest penetration, whereas AH Plus demonstrated the highest bond strength. The positive correlations observed suggest that dentinal tubule penetration may contribute to stronger bonding for specific sealers.

## 1. Introduction

One of the main goals of root canal treatment is to achieve a three‐dimensional filling of the root canal and establish a proper seal to prevent bacterial contamination [1, 2]. Gutta‐percha, the most commonly used core filling material, lacks adhesion to dentin and therefore, requires a sealer to improve sealing ability and ensure long‐term stability [3–7].

The primary function of a sealer is to enhance adhesion between root dentin and root canal filling materials by filling accessory canals and gaps between gutta‐percha cones and the root canal walls [8]. The sealer’s penetration into dentinal tubules creates a physical barrier that entombs any remaining bacteria [9] and increases the surface contact between the root canal walls and the filling materials, thereby improving the mechanical bond strength [10].

Besides penetration, the push‐out bond strength of sealers is a key property, as it determines their ability to resist dislodgement during function and retreatment [11, 12]. Therefore, evaluating both penetration and bond strength provides a more comprehensive understanding of sealer performance.

Recently, calcium silicate‐based (bioceramic) sealers such as Endoseal MTA and Endoseal TCS have been developed with improved bioactivity and handling compared to traditional epoxy resin‐based sealers like AH Plus [13–17].

MTA Fillapex is noted for its high radiopacity, low solubility, minimal expansion during setting, biocompatibility, effective sealing properties, and bactericidal action [18]. Additionally, MTA Fillapex releases calcium ions that stimulate tissue regeneration and healing [19].

Endoseal MTA is a pozzolan‐based bioceramic sealer made from calcium silicate, calcium aluminate, calcium aluminoferrite, and calcium sulfate [20]. It demonstrates excellent biocompatibility, sealing ability, and antibacterial properties [21]. Endoseal MTA is premixed in paste form and sets effectively in a moist canal environment [22, 23].

EndoSeal TCS (Maruchi) is derived from EndoSeal MTA and is composed of tricalcium silicate, phyllosilicate minerals, zirconium oxide, and dimethyl sulfoxide [24]. Unlike Endoseal MTA, EndoSeal TCS is purely tricalcium silicate‐based [24, 25].

However, limited evidence exists on how these newer sealers compare to resin‐based sealers regarding both penetration and bond strength, and whether a relationship exists between the two. Clinically, identifying such a relationship is important, since greater dentinal tubule penetration may enhance mechanical retention and contribute to long‐term sealing effectiveness, ultimately reducing the risk of reinfection and treatment failure. Therefore, this study aimed to evaluate dentinal tubule penetration and push‐out bond strength of AH Plus, MTA Fillapex, Endoseal MTA, and EndoSeal TCS (Maruchi) sealers and to assess the correlation between penetration depth and bond strength.

## 2. Materials and Methods

This study has been approved by the Ethics Committee of Babol University of Medical Sciences (IR.MUBABOL.REC. 1401.154).

### 2.1. Sample Selection and Preparation

Human single‐rooted anterior teeth were chosen for this in vitro study. The inclusion criteria for the study samples were: no root curvature, no fractures, closed apex, no internal or external resorption, and no history of prior root treatment. The sample size was determined to be 40, based on results from similar study comparing the penetration of resin‐based and bio‐ceramic sealers [1], with a desired study accuracy of 95% (*α* = 0.05) and power of 80% (*β* = 0.2).

A radiographic image was taken of each tooth to identify any root abnormalities. Teeth exhibiting abnormalities in the root or canal during radiography were excluded from the study. The samples were kept in a normal saline solution until all samples were collected.

### 2.2. Root Canal Filling

After preparing the access cavity and determining the working length, all teeth were instrumented using the rotary ProTaper system (Dentsply Sirona, Ballaigues, Switzerland) up to the F5 file as the master apical file. Canal patency was maintained throughout preparation using a #10 K‐file extended 0.5 mm beyond the apex between files. The canals were irrigated with 5 mL of 2.5% sodium hypochlorite solution, followed by a final rinse with 5 mL of 17% EDTA solution for 1 min, and then distilled water. After final rinse with distilled water, canals were dried with paper points until no visible moisture remained. The samples were then randomly assigned to four experimental groups (10 teeth in each group) based on the type of sealer used: AH Plus, MTA Fillapex, Endoseal MTA, and EndoSeal TCS (Maruchi).

To visible sealers under confocal laser scanning microscopy (CLSM), each sealer was mixed with 0.1% rhodamine B isothiocyanate fluorescent dye. For consistency, 10 parts of sealer were manually mixed with one part of rhodamine B isothiocyanate dye powder using a sterile glass slab and spatula until homogeneity was achieved. The root canals were then filled with gutta‐percha and the respective sealers using the single cone technique. To apply the sealer, an F5 gutta‐percha cone coated with the sealer was placed into the canal using a pumping method. After the canal walls were coated with sealer, additional gutta‐percha was inserted to the working length, ensuring that excess sealer was expelled from the access cavity to guarantee adequate sealer presence inside the canal. The access cavities were then sealed with Cavit and the samples were incubated at 37°C and 100% humidity for 1 week [26].

Subsequently, three slices were obtained from each tooth—one from the coronal third, one from the middle third, and one from the apical third—perpendicular to the root’s longitudinal axis, each with a thickness of 1.0 ± 0.1 mm. These slices were cut using a sectioning machine (Nemo, Mashhad, Iran) equipped with a water coolant and a 0.3 mm thick blade oriented perpendicular to the tooth’s longitudinal axis.

### 2.3. CLSM

The same slices were used first for CLSM analysis and subsequently for push‐out testing to ensure direct comparison. The extent and percentage of sealer penetration into dentinal tubules were assessed using CLSM (Leica TCS‐SPE, Leica Microsystems, Wetzlar, Germany) on the prepared sections (Figure 1). Initially, all samples were mounted on glass slides and examined with a Leica TCS‐SPE confocal microscope and images were captured by 40× magnification. To evaluate the images, the method described by Gharib et al. [27] was employed, and sealer penetration was measured using Adobe Photoshop 7.0 software (Adobe Systems, San Jose, CA, USA). The lasso tool was utilized to select and measure the canal area. The areas along the canal walls where the sealer had penetrated dentinal tubules were identified and measured similarly. The distances along the canal circumference were used to calculate the percentage of sealer penetration in those areas. Using the ruler tool in the image analysis software, sealer penetration was measured at four standardized points (mesial, distal, buccal, and lingual) in each section. Measurements were taken from the canal wall, and the average of these measurements was reported as the extent of sealer penetration for that sample.

Figure 1The microscopy view of sealer penetration into dentinal tubules, observed using a confocal laser scanning microscopy (magnification 40×). Panels (A) through (D) show the following sealers: (A) AH Plus, (B) MTA Fillapex, (C) Endoseal MTA, and (D) Endoseal TCS.

**Figure:**
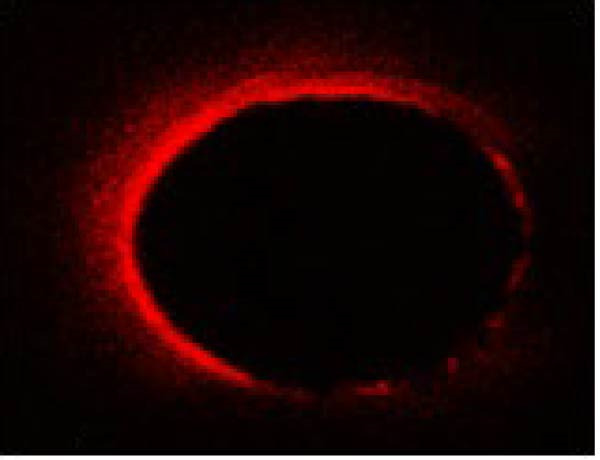
(A)

**Figure:**
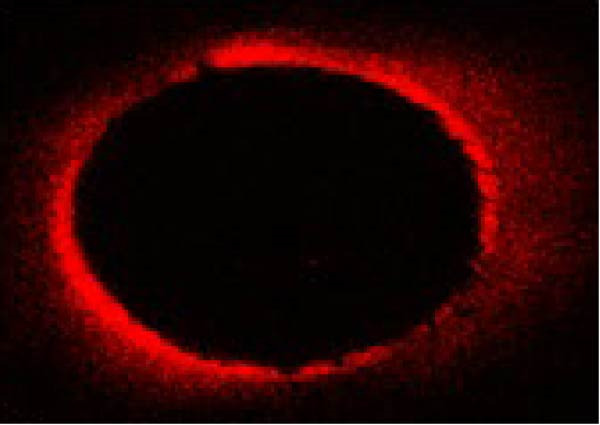
(B)

**Figure:**
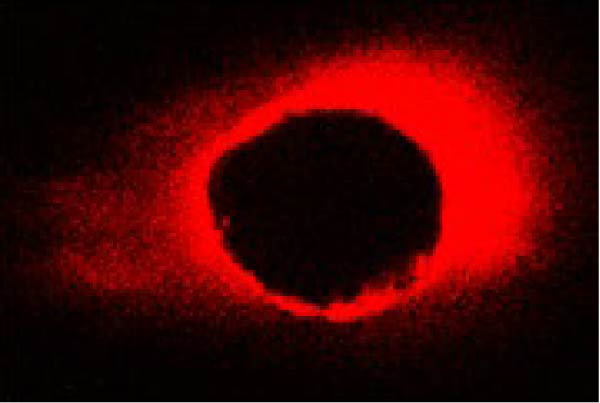
(C)

**Figure:**
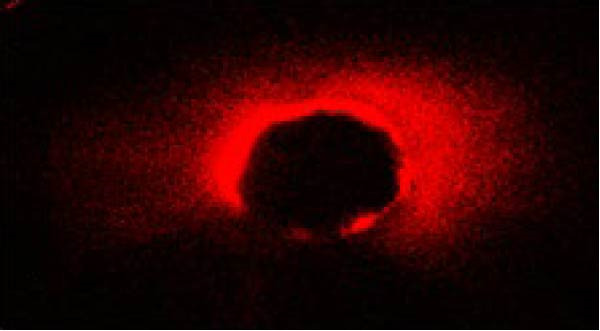
(D)

### 2.4. Push‐out Bond Strength Test

To measure the bond strength of sealers to dentin walls, a push‐out bond strength test was conducted on the same slices, from the apical to the coronal level. A plunger with varying diameters (ranging from 0.5 to 1 mm and depending on the canal diameter in the relevant section) was used, applying a load at a speed of 0.5 mm/min with a 200 kg load cell on a universal testing machine (Koopa, Sari, Iran). Prior to testing, the cross‐sectional surface of each slice was examined with a stereomicroscope (Dwinter, Milan, Italy), and the canal diameter was measured from both sides to calculate the contact area between the sealer and dentin walls. The maximum force at failure was recorded, and push‐out bond strength was calculated with the following formula:

Bond strength =Force at failure N/bonded area mm2.

### 2.5. Failure Mode Analysis

The samples were subsequently examined under a stereomicroscope to classify the type of failure as adhesive, cohesive, or mixed [8, 28].

### 2.6. Statistical Analysis

Data were analyzed using SPSS software (IBM, New York, USA) Version 22. Data normality was assessed with Shapiro–Wilk test and homogeneity with Levene’s test. Since data were parametric, one‐way and two‐way ANOVA with Tukey post hoc were applied. Pearson correlation was used to assess the correlation between penetration depth and bond strength. Significance was set at *p*  < 0.05.

## 3. Results

Table 1 presents the mean extent and percentage of sealer penetration into dentinal tubules, as well as the push‐out bond strength of the sealers studied. Statistically significant differences were observed in both the extent and the percentage of penetration into dentinal tubules, and the bond strength among the four sealers (All *p*  < 0.001).

**Table 1:** Mean extent and percentage of sealer penetration into dentinal tubules and the push‐out bond strength of the sealers studied.

Sealer type	Extent of penetration (µm)		Percentage of penetration (%)		Push‐out bond strength (MPa)	
	Mean	SD	Mean	SD	Mean	SD
AH plus	167.17	69.5	75.83	10.96	7.11	1.63
MTA Fillapex	452.87	156.09	75.5	19.16	1.73	0.63
Endoseal MTA	1077.87	466.06	87.2	11.52	6.35	1.8
Endoseal TCS	905.8	391.69	91.23	10.07	6.29	1.84
*p*‐Value^a^	**<0.001**		**<0.001**		**<0.001**	

*Note:* The bold values indicate statistical significance.

^a^One‐way ANOVA.

Table 2 presents the pairwise comparisons of sealer groups. Endoseal MTA and Endoseal TCS showed significantly greater dentinal tubule penetration depth and percentage compared to AH Plus and MTA Fillapex (*p*  < 0.001). MTA Fillapex demonstrated significantly lower bond strength than the other three sealers (*p*  < 0.001). No significant difference in bond strength was found between AH Plus, Endoseal MTA, and Endoseal TCS (*p*  > 0.05).

**Table 2:** Pairwise comparisons of the mean extent and percentage of sealer penetration into dentinal tubules and the push‐out bond strength of the sealers studied.

Variables	Sealer type		Mean difference	95% CI		*p*‐Value^a^
				Lower	Upper	
Depth of penetration (µm)	AH plus	MTA Fillapex	−285.7	−498.49	−72.91	**0.004**
		Endoseal MTA	−910.7	−1123.49	−697.91	**<0.001**
		Endoseal TCS	−738.63	−951.42	−525.85	**<0.001**
	MTA Fillapex	Endoseal MTA	−625	−837.79	−412.21	**<0.001**
		Endoseal TCS	−452.93	−665.72	−240.15	**<0.001**
	Endoseal MTA	Endoseal TCS	172.07	−40.72	384.85	0.157

Percentage of penetration (%)	AH plus	MTA Fillapex	0.33	−8.71	9.37	>0.999
		Endoseal MTA	−11.37	−20.41	−2.33	**0.007**
		Endoseal TCS	−15.4	−24.44	−6.36	**<0.001**
	MTA Fillapex	Endoseal MTA	−11.7	−20.74	−2.66	**0.005**
		Endoseal TCS	−15.73	−24.77	−6.69	**<0.001**
	Endoseal MTA	Endoseal TCS	−4.03	−13.07	5.01	0.651

Push‐out bond strength (MPa)	AH plus	MTA Fillapex	5.38	4.34	6.43	**<0.001**
		Endoseal MTA	0.76	−0.29	1.8	0.24
		Endoseal TCS	0.82	−0.23	1.87	0.178
	MTA Fillapex	Endoseal MTA	−4.62	−5.67	−3.58	**<0.001**
		Endoseal TCS	−4.56	−5.61	−3.52	**<0.001**
	Endoseal MTA	Endoseal TCS	0.06	−0.98	1.11	0.999

*Note:* The bold values indicate statistical significance.

^a^Tukey post hoc.

Table 3 shows the mean penetration depth, penetration percentage, and push‐out bond strength of the sealers across coronal, middle, and apical thirds. For all sealers, both penetration depth and percentage decreased significantly from coronal to apical regions (*p*  < 0.05), except for Endoseal TCS, which maintained comparable penetration percentages across thirds. Push‐out bond strength of AH Plus and MTA Fillapex remained relatively stable across root levels, whereas Endoseal MTA and Endoseal TCS exhibited a significant reduction in bond strength from coronal to apical thirds (*p*  < 0.001).

**Table 3:** Mean extent and percentage of sealer penetration into dentinal tubules and the push‐out bond strength of the studied sealers by root thirds.

Variables	Sealer type	Root thirds						*p*‐Value^a^
		Coronal		Middle		Apical		
		Mean	SD	Mean	SD	Mean	SD	
Depth of penetration (µm)	AH plus	229.6	67.99	155	50.99	116.9	23.2	**<0.001**
	MTA Fillapex	579.2	107.82	485.2	115.67	294.2	81.44	**<0.001**
	Endoseal MTA	1483.7	304.74	1171.8	328.12	578.1	170.42	**<0.001**
	Endoseal TCS	1210	314.82	1034.1	225.62	473.3	124.01	**<0.001**
	*p*‐Value^a^	**<0.001**		**<0.001**		**<0.001**		—

Percentage of penetration (%)	AH plus	83	8.73	77.2	10.46	67.3	7.83	**0.002**
	MTA Fillapex	88.4	11.07	79.4	17.24	58.7	15.76	**<0.001**
	Endoseal MTA	94.8	7.16	85.2	12.75	81.6	10.49	**0.024**
	Endoseal TCS	96.3	4.03	88.7	9.15	88.7	13.57	0.15
	*p*‐Value^a^	**<0.003**		0.184		**<** **0.001**		—

Push‐out bond strength (MPa)	AH plus	8.02	1.4	6.42	1.29	6.89	1.84	0.072
	MTA Fillapex	1.68	0.73	1.77	0.64	1.72	0.56	0.95
	Endoseal MTA	7.92	1.43	6.26	1.07	4.87	1.41	**<0.001**
	Endoseal TCS	8.09	1.4	6.02	1.39	4.76	0.87	**<0.001**
	*p*‐Value^a^	**<0.001**		**<0.001**		**<0.001**		—

*Note:* The bold values indicate statistical significance.

^a^One‐way ANOVA.

Table 4 presents pairwise comparisons among root thirds for each sealer. The coronal third consistently demonstrated significantly greater penetration depth and percentage compared to the apical third for all sealers (*p*  < 0.05). Differences between middle and apical thirds were also significant in most cases, especially for MTA Fillapex, Endoseal MTA, and Endoseal TCS. Regarding bond strength, significant differences between coronal and apical thirds were evident for Endoseal MTA and Endoseal TCS, while AH Plus and MTA Fillapex showed no statistically significant variation across thirds.

**Table 4:** Pairwise comparison of the mean extent and percentage of sealer penetration into dentinal tubules and the push‐out bond strength of each studied sealer by root thirds.

Variables	Sealer type	Root third		Mean difference	95% CI		*p*‐Value^a^
					Lower	Upper	
Depth of penetration (µm)	AH plus	Coronal	Middle	74.6	16.42	132.78	**0.01**
			Apical	112.7	54.52	170.88	**<0.001**
		Middle	Apical	38.1	−20.08	96.28	0.253
	MTA Fillapex	Coronal	Middle	94	−19.87	207.87	0.12
			Apical	285	171.13	398.87	**<0.001**
		Middle	Apical	191	77.13	304.87	**0.001**
	Endoseal MTA	Coronal	Middle	311.9	5.16	618.64	**0.046**
			Apical	905.6	598.86	1212.34	**<0.001**
		Middle	Apical	593.7	286.96	900.44	**<0.001**
	Endoseal TCS	Coronal	Middle	175.9	−84.45	436.25	0.233
			Apical	736.7	476.35	997.05	**<0.001**
		Middle	Apical	560.8	300.45	821.15	**<0.001**

Percentage of penetration (%)	AH plus	Coronal	Middle	5.8	−4.26	15.86	0.341
			Apical	15.7	5.64	25.76	**0.002**
		Middle	Apical	9.9	−0.16	19.96	0.054
	MTA Fillapex	Coronal	Middle	9	−7.55	25.55	0.381
			Apical	29.7	13.15	46.25	**<0.001**
		Middle	Apical	20.7	4.15	37.25	**0.012**
	Endoseal MTA	Coronal	Middle	9.6	−1.92	21.12	0.116
			Apical	13.2	1.68	24.72	**0.022**
		Middle	Apical	3.6	−7.92	15.12	0.721
	Endoseal TCS	Coronal	Middle	7.6	−3.19	18.39	0.207
			Apical	7.6	−3.19	18.39	0.207
		Middle	Apical	0	−10.79	10.79	>0.999

Push‐out bond strength (MPa)	AH plus	Coronal	Middle	1.6	−0.09	3.29	0.67
			Apical	1.14	−0.55	2.83	0.235
		Middle	Apical	−0.46	−2.15	1.23	0.781
	MTA Fillapex	Coronal	Middle	−0.09	−0.81	0.63	0.945
			Apical	−0.04	−0.76	0.67	0.987
		Middle	Apical	0.05	−0.67	0.77	0.984
	Endoseal MTA	Coronal	Middle	1.67	0.21	3.12	**0.023**
			Apical	3.06	1.6	4.52	**<0.001**
		Middle	Apical	1.39	−0.06	2.85	0.063
	Endoseal TCS	Coronal	Middle	2.07	0.69	3.45	**0.003**
			Apical	2.33	1.95	4.71	**<0.001**
		Middle	Apical	1.26	−0.12	2.64	0.078

*Note:* The bold values indicate statistical significance.

^a^Tukey post‐hoc.

**Figure 2:**
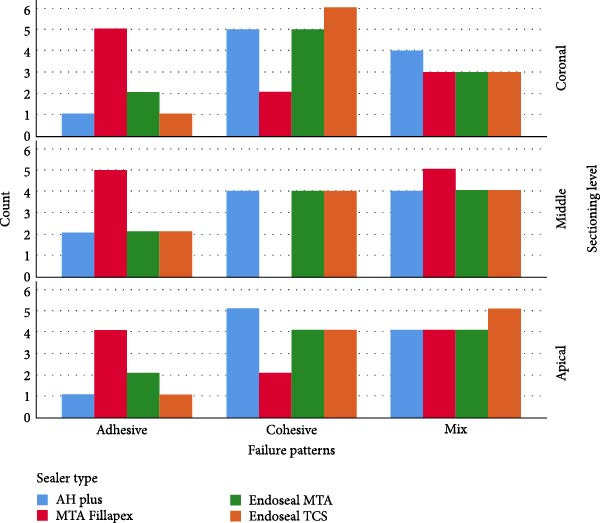
Failure patterns by sealer type and root thirds.

Figure 2 present the frequency of failure patterns for each sealer, categorized by the location of the root sections.

Table 5 summarizes the correlations between dentinal tubule penetration (depth and percentage) and push‐out bond strength. For AH Plus, a weak but significant positive correlation was observed only between bond strength and penetration percentage (*r* = 0.366 and *p* = 0.046). MTA Fillapex showed no significant correlation with either penetration depth or percentage. Endoseal MTA exhibited significant positive correlations with both penetration depth (*r* = 0.448 and *p* = 0.013) and penetration percentage (*r* = 0.572 and *p* = 0.001). Endoseal TCS demonstrated a significant correlation with penetration depth (*r* = 0.57 and *p* = 0.001), but not with penetration percentage.

**Table 5:** Correlation between the mean push‐out bond strength and mean extent and percentage of sealer penetration into dentinal tubules.

Sealer type	Push‐out bond strength	Depth of penetration	Percentage of penetration
AH plus	Correlation coefficient (*r*)	0.287	**0.366**
	*p*‐Value^a^	0.124	**0.046**

MTA Fillapex	Correlation coefficient (*r*)	0.005	0.252
	*p*‐Value^a^	0.98	0.179

Endoseal MTA	Correlation coefficient (*r*)	**0.448**	**0.572**
	*p*‐Value^a^	**0.013**	**0.001**

Endoseal TCS	Correlation coefficient (*r*)	**0.57**	0.294
	*p*‐Value^a^	**0.001**	0.115

*Note:* The bold values indicate statistical significance.

^a^Pearson’s correlation.

## 4. Discussion

In this study, the penetration of four endodontic sealers into dentinal tubules and their push‐out bond strength to root dentin were evaluated, and the correlation between these two variables was analyzed. Endoseal MTA and Endoseal TCS demonstrated the greatest penetration, whereas AH Plus and MTA Fillapex showed the least. Despite its lower penetration, AH Plus exhibited the highest bond strength, while MTA Fillapex had the lowest. Importantly, positive correlations between penetration and bond strength were identified for Endoseal MTA and Endoseal TCS. These findings indicate that calcium silicate‐based sealers may achieve both deeper tubule penetration and favorable adhesion, supporting their clinical applicability.

The extent of sealer penetration into dentinal tubules is influenced by several factors, including particle size, solubility, viscosity, and the root canal filling technique [29]. The higher penetration observed with Endoseal MTA and Endoseal TCS sealers can be attributed to their hydrophilicity and smaller particle size, whereas the lower penetration of AH Plus is due to its hydrophobic nature [30, 31]. Additionally, previous studies have demonstrated that using the warm vertical technique to fill the root canal, combined with an increase in the viscosity of AH Plus sealer, enhances its penetration into dentinal tubules [32]. Since the root canal in the present study was filled using the single cone technique, it is expected that the penetration of AH Plus sealer would be less affected by this filling method. Although MTA Fillapex has high fluidity, its longer setting time, due to the high resin content, results in significant volumetric contraction [28]. Therefore, it is anticipated that MTA Fillapex’s penetration into dentinal tubules would be less compared to other bioceramic sealers. Additionally, the penetration of all four sealers into dentinal tubules significantly decreased from the coronal to the apical region. This finding is consistent with the fact that the density and diameter of dentinal tubules decrease from the coronal to the apical part of the root [33].

Based on the results of the current study, Endoseal TCS and Endoseal MTA exhibited the highest percentage of penetration into dentinal tubules, while MTA Fillapex and AH Plus showed the lowest. Kuçi et al. [34] found that MTA Fillapex had a higher penetration percentage compared to AH Plus, while Tuncer et al. [2] reported no statistically significant difference in penetration percentage between AH Plus and MTA Fillapex sealers concerning bond strength, which aligns with the present study’s findings. Additionally, Kaur et al. [35] reported significantly higher penetration into dentinal tubules for Endoseal MTA compared to AH Plus, which is consistent with our results.

Furthermore, Song and Yang [36] demonstrated that Endoseal MTA sealer, when using the single cone technique, exhibited higher penetration compared to AH Plus sealer with the single cone technique, but lower penetration compared to AH Plus sealer with the continuous wave technique. Since only the single cone method was used in the present study, a comparison with the continuous wave technique was not possible. However, the findings of the present study are consistent with those of Song and Yang regarding the comparison of Endoseal MTA and AH Plus sealers using the single cone method.

On the other hand, Kim et al. [37] reported that the penetration of AH Plus sealer was significantly higher compared to Endoseal MTA, which contradicts the findings of the present study. In their study, the continuous wave technique was used for AH Plus samples, while the single cone technique was used for Endoseal MTA samples. This difference in root canal filling methods likely explains the discrepancy between the results of the two studies.

The hydrophilic nature and smaller particle size of Endoseal MTA and Endoseal TCS sealers not only result in increased sealer penetration into dentinal tubules but also likely allow these sealers to penetrate a greater number of tubules, thereby increasing the percentage of penetration. Conversely, the hydrophobic and resinous nature of AH Plus sealer, combined with the single cone filling technique, leads to fewer dentinal tubules being penetrated, thus reducing its penetration percentage. Furthermore, a study observed that the surface characteristics of MTA Fillapex sealer were similar to those of AH Plus in scanning electron microscopy (SEM) images [38]. Given the resin content and volumetric contraction of MTA Fillapex sealer, its penetration percentage is expected to be lower compared to other sealers.

Based on the results, the penetration percentage of Endoseal MTA, AH Plus, and MTA Fillapex sealers significantly decreased from the coronal to the apical region. However, this decrease was not statistically significant for Endoseal TCS sealer. Given that the density and diameter of dentinal tubules decrease from the coronal to the apical region [33], it is expected that the penetration percentage of sealers into dentinal tubules would also decrease. No previous studies have specifically examined the properties of Endoseal TCS. While Endoseal TCS is similar to Endoseal MTA, its silicate component is tricalcium silicate instead of pozzolan cement [26, 39]. The presence of tricalcium silicate in Endoseal TCS may result in increased water affinity, and consequently, increased penetration even in the apical regions. However, this hypothesis requires further investigation in future studies.

The results of the present study showed that the lowest push‐out bond strength was associated with MTA Fillapex sealer. The other three sealers exhibited similar bond strengths, but the bond strength of MTA Fillapex was significantly lower. Additionally, the push‐out bond strength of AH Plus and MTA Fillapex sealers remained consistent throughout the root canal length, whereas the bond strength of Endoseal MTA and Endoseal TCS decreased significantly from the coronal to the apical region.

The positive associations found for Endoseal MTA and Endoseal TCS (and to a lesser degree AH Plus), contrasted with the absence of correlation for MTA Fillapex, and the clinical implications of these differences. Given the relatively high linear correlation between push‐out bond strength and penetration into dentinal tubules for Endoseal MTA and Endoseal TCS sealers in this study, a decrease in penetration into dentinal tubules is expected to reduce the push‐out bond strength of these two sealers. These sealers form a micromechanical bond with dentin due to the presence of a calcium silicate base [40]. Furthermore, considering that these two sealers had the highest degree of penetration into dentinal tubules, their high push‐out bond strength is justified.

The results of this study showed that the failure patterns in samples related to MTA Fillapex sealer were predominantly adhesive and mixed, while cohesive and mixed fractures were more common in samples with the other sealers. Based on the push‐out bond strength results, it can be expected that the fracture patterns of AH Plus, Endoseal MTA, and Endoseal TCS sealers are similar, with adhesive failures being less likely to occur. Additionally, due to the low push‐out bond strength of MTA Fillapex sealer, a higher percentage of adhesive fractures is justified.

Silva et al. reported that the displacement resistance was significantly higher in AH Plus sealer compared to MTA Fillapex. They noted that this finding has been well demonstrated in previous texts, consistent with our results [28]. In another study, Silva et al. [26] examined the push‐out bond strength of AH Plus, MTA Fillapex, and Endoseal. According to their results, AH Plus had the highest push‐out bond strength, while MTA Fillapex had the lowest. The higher bond strength of Endoseal compared to MTA Fillapex observed in their study aligns with the present study’s findings. However, there was a statistically significant difference in bond strength between AH Plus and Endoseal in their study. They examined only six teeth and focused solely on the middle third of the root. Additionally, instead of using the root canal for assessing push‐out bond strength, they created three holes in the dentin around the canal and used these for testing. Since the diameter of dentinal tubules decreases away from the root canal due to dentin deposition, the degree of penetration and bond strength achieved in their experiment may differ from actual values.

Sarrafan et al. demonstrated that AH26 exhibited a similar push‐out bond strength to Endoseal MTA, and the bond strength of both was significantly higher than that of MTA Fillapex, consistent with the findings of the present study. Additionally, MTA Fillapex had a higher number of adhesive fractures compared to AH26 and Endoseal MTA, with no cohesive fractures observed [41]. Furthermore, other studies corroborate these findings [2, 42]. However, no studies on the push‐out bond strength of Endoseal TCS sealer were found.

The bond strength of AH Plus sealer can be attributed to the formation of a covalent bond between epoxy rings and amino groups in the dentin collagen network [2]. Additionally, previous studies have cited factors such as lower polymerization stress and shrinkage, long‐term dimensional stability, molecular adhesion, and the potential for greater penetration into irregular dentin surfaces as reasons for the higher bond strength of AH Plus sealer [26]. Consequently, it is expected that among the sealers used, AH Plus sealer would exhibit high push‐out bond strength, unaffected by its position along the root canal.

Although MTA Fillapex sealer contains a large volume of resin, it does not form a covalent bond like AH Plus sealer. Additionally, a 1:1 ratio of salicylate resin and MTA is necessary for the proper setting of MTA Fillapex sealer. However, studies have shown that the salicylate ratio in MTA Fillapex is higher, which increases its setting time. During setting, sealers containing salicylate resin experience volumetric shrinkage, leading to gap formation between the root canal and the sealer. Thus, a higher proportion of salicylate in MTA Fillapex sealer results in decreased bond strength [26]. Consequently, the push‐out bond strength of MTA Fillapex sealer is significantly lower compared to other sealers.

### 4.1. Limitations and Suggestions for Further Studies

This study has some limitations. As an in vitro investigation, it may not fully reproduce the clinical environment, including variations in intraoral temperature, humidity, and long‐term functional stresses. In clinical situations, periapical tissues may present acidic pH, which could impair setting, adhesion, and interfacial stability of calcium silicate‐based sealers. Our findings of penetration and bond strength should be interpreted in light of studies such as Özel and Erişen [43], indicating environmental pH can modulate performance. Only the single‐cone obturation technique was evaluated, and different techniques may influence both penetration and bond strength. In addition, CLSM with fluorescent dye, while widely used, may not perfectly replicate sealer behavior in vivo. Future studies should assess long‐term bond durability under functional loading, explore additional obturation techniques, and include in vivo evaluations to confirm the clinical significance of the observed correlations.

## 5. Conclusions

According to the results of the present study, Endoseal MTA and Endoseal TCS sealers exhibited the highest depth and percentage of penetration into dentinal tubules, while AH Plus and MTA Fillapex sealers showed the lowest penetration. However, AH Plus sealer demonstrated the highest push‐out bond strength, comparable to that of Endoseal MTA and Endoseal TCS sealers. In contrast, MTA Fillapex showed the lowest push‐out bond strength. Significant positive correlations were found between dentinal tubule penetration and push‐out bond strength for certain sealers, highlighting the clinical relevance of sealer penetration in improving adhesion.

## Disclosure

This manuscript is the result of the research project approved by the Vice‐Chancellor of Research and Technology of Babol University of Medical Sciences #724134705.

## Conflicts of Interest

The authors declare no conflicts of interest.

## Funding

This research did not receive any specific grant from funding agencies in the public, commercial, or not‐for‐profit sectors.

## Data Availability

The data that support the findings of this study are available from the corresponding author upon reasonable request.
